# Could serum albumin value and thrombocyte/lymphocyte ratio be an important prognostic factor in determining the severity of COVID 19?

**DOI:** 10.3906/sag-2008-285

**Published:** 2021-06-28

**Authors:** Emel UYAR, Alparslan MERDİN, Serdar YAMANYAR, Mehmet Can EZGÜ, Cumhur ARTUK, Gürhan TAŞKIN, Yakup ARSLAN, Serkan CERİTLİ

**Affiliations:** 1 Department of Critical Care Medicine, University of Health Sciences, Gülhane Education Research Hospital, Ankara Turkey; 2 Department of Hematology, University of Health Sciences, Gülhane Education Research Hospital, Ankara Turkey; 3 Department of Neurosurgery, University of Health Sciences, Gülhane Education Research Hospital, Ankara Turkey; 4 Department of Infection, University of Health Sciences, Gülhane Education Research Hospital, Ankara Turkey; 5 Department of Chest Diseases, University of Health Sciences, Gülhane Education Research Hospital, Ankara Turkey; 6 Department of Emergency Medicine, University of Health Sciences, Gülhane Education Research Hospital, Ankara Turkey

**Keywords:** Severity of COVID 19, platelet lenfosit ratio, serum albumin value

## Abstract

**Background and aim:**

Creating potential clinical markers for risk assessment in patients with COVID-19 continues to be an area of interest. In this study, we aimed to evaluate whether serum albumin level and thrombocyte/lymphocyte ratio are related to the severity of the disease.

**Materials and methods:**

The patients were divided into two groups according to the severity of disease. Demographic data, serum albumin value, lymphocyte count, TLO-1 values (thrombocyte/lymphocyte ratio-1), the highest thrombocyte count during hospitalization, TLO-2 (thrombocyte/lymphocyte ratio-2) values formed by the highest thrombocyte count, were recorded.

**Results:**

There was no statistically significant differences (P > 0.05) in terms of sex, thrombocyte count at the time of admission, and highest thrombocyte count during hospital follow-up. There were statistically significant differences in terms of age, comorbidity, lymphocyte value at the time of hospitalization, lymphocyte count during hospital follow-up, TLO 1, TLO 2, and serum albumin values between the groups. The ICU group were found to be older, had higher rates of comorbidity, lower lymphocyte values, higher TLO 1-2, and lower serum albumin levels (P < 0.05).

**Conclusion:**

TLO-2 ratio above 260 and lymphocyte level below 1 103 cells/μL, would be a predictor of further intensive care unit need.

## 1. Introduction

The COVID-19 disease was seen to start with the occurrence of idiopathic pneumonia cases in China in December 2019 [1,2].World Health Organization (2020). Novel Coronavirus – China [online]. Website http://www.who.int/csr/don/12-january-2020-novel-coronavirus-china/en/ [accesed 00 Month Year]. At the end of 2019 in Wuhan, China, the COVID-19 virus spread rapidly all over China and then to many parts of the world [3]. The COVID-19 cases in many countries apart from China, where the first epidemic started, were observed due to the spread and severity of the virus. Therefore, The COVID-19 was defined as a global epidemic (pandemic) on March 11, 2020.World Health Organization (2020). Name of source [online]. Website https://www.who.int/docs/defaultsource/coronaviruse/situation-reports/2020022 sitrep-32-covid-19.pdf [accessed 00 Month Year].  While clinical signs of fever, weakness, cough, and shortness of breath can be observed in the patients, laboratory findings may be normal, as well as decreased leukocyte count, decreased lymphocyte count, thrombocytopenia, increased transaminases, increased lactate dehydrogenase (LDH), and creatine kinase-myoglobin elevation [4]. However, not much is known about clinical markers for risk of worse prognosis in patients with COVID-19. Currently, there is no specific treatment for COVID-19. Therefore, the evaluation of disease severity, course, and prognosis becomes increasingly important [5].

In this study, we aimed to evaluate whether serum albumin level, thrombocyte count, lymphocyte count and thrombocyte/lymphocyte ratio were related to the severity of the disease.

## 2. Materials and methods

Our study was a retrospective study and was planned on patients with positive COVID-19 PCR tests who applied to our hospital on March, April, May 2020. The study was conducted after the approval of the Gülhane Education and Research Hospital Ethics Committee, with project number 2020-10. The laboratory tests of 63 patients with positive COVID-19 PCR test, 33 being followed in the intensive care unit, and 30 patients in the inpatient unit of the Gülhane Education and Research Hospital, were scanned through the hospital automation system. Signs of COVID-19 infection may be dyspnea and respiratory distress. Patients with a respiratory rate > 30 /min, PaO_2_ / FiO_2_ < 300, SpO_2_ < 90, or PaO_2_ < 70 despite 5 L/m oxygen therapy were evaluated in terms of intensive care admission in the common routine practice of our hospital. We formed 2 groups according to the severity of disease; those who were followed up in the intensive care unit (severe COVID-19 patients), and those deemed appropriate for follow-up in the inpatient unit (moderate and mild COVID-19 patients). Patients under 18 years of age, patients with chronic liver failure, hematological malignancy or chronic hematological disease (immune thrombocytopenic purpura, paroxysmal nocturnal hemoglobinuria, thrombotic thrombocytopenic purpura, aplastic anemia, immune or nonimmune secondary thrombocytopenia before COVID-19), malignancy diseases with nonremission status, and patients who were receiving chemotherapy and radiotherapy were not included in the study.

The demographic data, comorbidities, and laboratory values of 63 patients who met the inclusion criteria were recorded. Peripheral blood samples of the patients were analyzed as a conventional medical approach at admission and during hospitalization. Demographic data, serum albumin value during hospitalization, lymphocyte count, TLO-1 values (thrombocyte/lymphocyte ratio-1), the highest thrombocyte count during hospitalization, the highest thrombocyte count, and TLO-2 values (thrombocyte/lymphocyte ratio-2) formed by the highest thrombocyte count was recorded. TLO-1 and TLO-2 values were calculated by using laboratory data. The Apache scores of the patients followed in the intensive care unit were recorded. The relationship between independent variables and the severity of COVID-19 disease was investigated. 

All statistical analysis was performed using IBM SPSS version 25.0 (IBM Corp., Armonk, NY, USA) and MedCalc 15.8 (MedCalc Software Ltd, Ostend, Belgium) statistical package programs. While evaluating the study data, besides descriptive statistical methods (frequency, percentage, mean, standard deviation, median, min-max), the chi-square test was also used to compare qualitative data. The compliance of the data to normal distribution was evaluated by Kolmogorov–Smirnov and Shapiro–Wilk tests. In the study, Independent samples t-test was used for intergroup comparisons of normally distributed data, paired samples t-test for comparing repetitive measurements, Mann–Whitney U test for intergroup comparisons of nonnormally distributed data, and Wilcoxon signed ranks test for comparing repetitive measurements. The ROC curve (receiver operating characteristic) method was used to determine the distinctiveness of the variables. Correlations between variables were evaluated using the Pearson correlation test. Values with a probability (P) of < 0.05 were considered statistically significant, and differences between groups.

## 3. Results

A total of 63 patients (30 COVID intensive care unit patients, and 33 COVID-1 inpatient unit patients) were included in the study. The patients included in the study were 29 females and 34 males. The average age of the patients with positive COVID-19 who were followed up was 53 years (Table 1).

**Table 1:** Characteristics of the patients.

		n = 63	%
Group	Inpatient unit	33	52.4
	ICU	30	47.6
Sex	Female	29	46.0
	Male	34	54.0
Age (year)*		53.4 ± 19.4	53.0 (18.0–88.0)
Comorbidities	No	33	52.4
	Yes	30	47.6

When both groups (inpatient unit and ICU unit) were compared; there was no statistically significant differences (P > 0.05) in terms of sex, thrombocyte I (thrombocyte count at the time of admission), and thrombocyte II (highest thrombocyte count during hospital follow-up). At the same time, there were statistically significant differences in terms of age, comorbidity, lymphocyte I (lymphocyte value at the time of hospitalization), lymphocyte II (lymphocyte count during hospital follow-up), TLO 1 (thrombocyte/lymphocyte ratio at the time of hospital admission), TLO 2 (highest thrombocyte during hospital follow-up), and serum albumin values between the groups (Table 2).

**Table 2:** Comparisons between groups.

		Inpatient unit(n = 33)	ICU (n = 30)	P
Sex1	Female	15 (45.5%)	14 (46.7%)	1.000 a
	Male	18 (54.5%)	16 (53.3%)	
Age (year)2	General	40.5 ± 13.5	67.6 ± 14.4	0.000 b
	Female	36.5 ± 1.5	67.1 ± 16.0	0.000 b
	Male	43.9 ± 10.9	67.9 ± 13.3	0.000 b
	P	0.117 b	0.883 b	
Comorbidities 1	No	24 (72.7%)	9 (30.0%)	0.002 a
	Yes	9 (27.3%)	21 (70.0%)	
Thrombocytes 3	I	195.0 (155.5–220.5)	189.5 (152.3–246.3)	0.836 c
	II	221.0 (195.5–297.0)	307.5 (223.0–374.3)	0.070 c
	P	0.000 d	0.000d	
Lymphocytes 3	I	1.4 ± 0.5	1.1 ± 0.7	0.012 b
	II	1.6 ± 0.5	1.2 ± 0.8	0.013 b
	P	0.005 e	0.386 e	
TLO 3	I	159.0 (115.0–205.5)	229.0 (151.5–309.3)	0.014 c
	II	170.0 (122.8–232.0)	329.5 (178.0–598.3)	0.000 c
	P	0.323 d	0.002 d	
Albumin 2		4.0 ± 0.3	3.1 ± 0.3	0.000 b
Apache 2		--	40.1 ± 16.7	--

**Figure 1:**
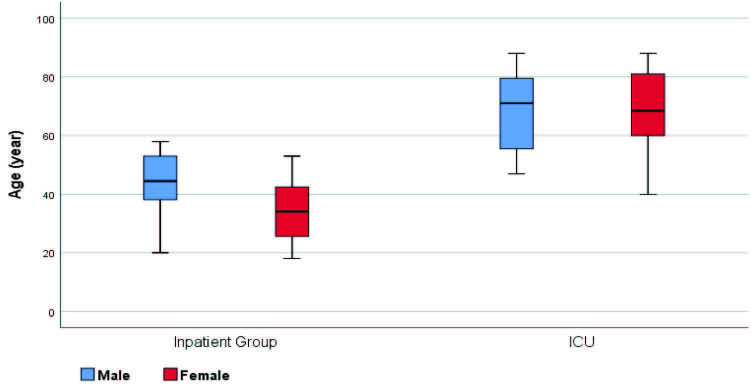
In-group and between-group comparisons/age.

**Figure 2:**
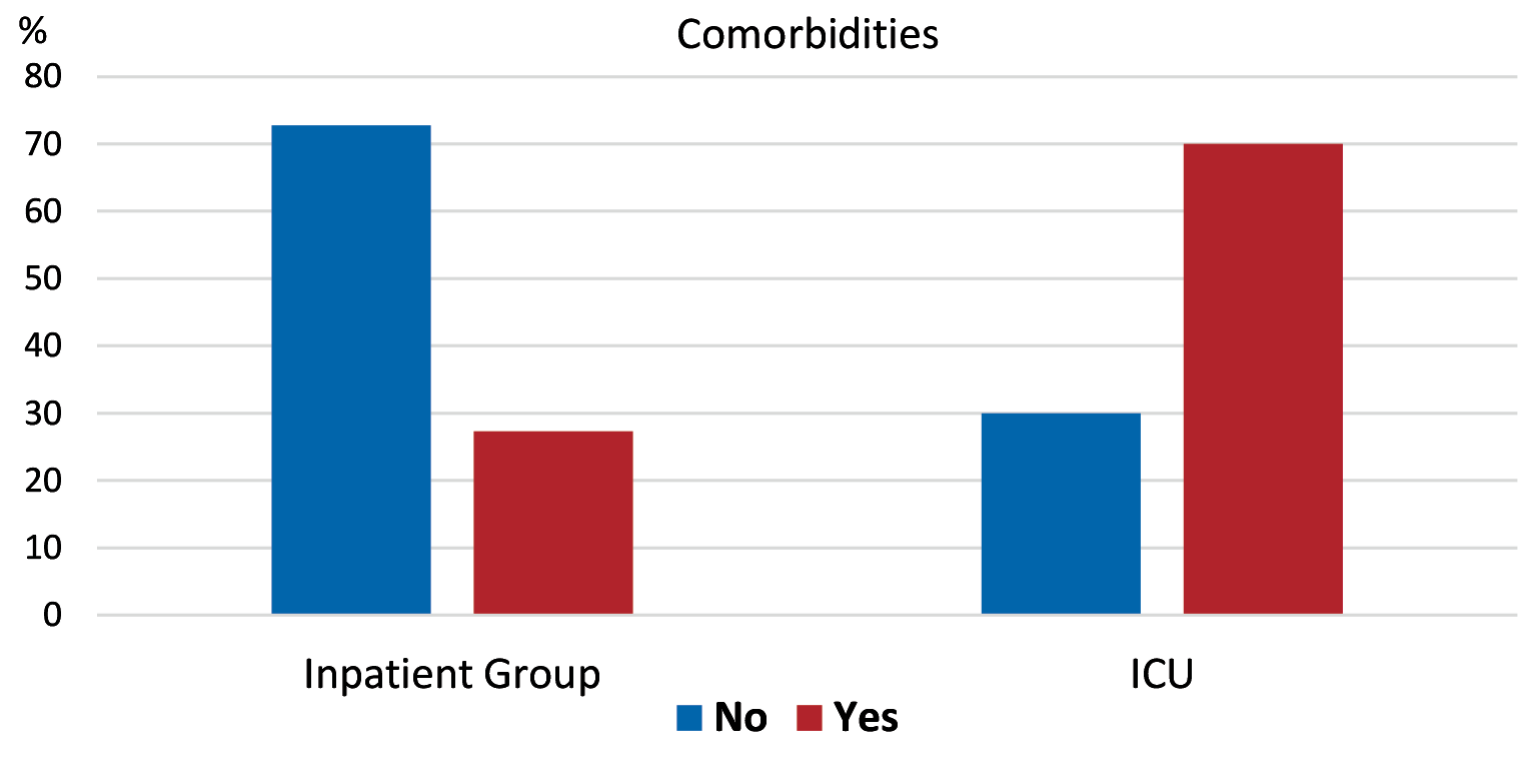
In-group and intergroup comparisons/comorbidities.

**Figure 3:**
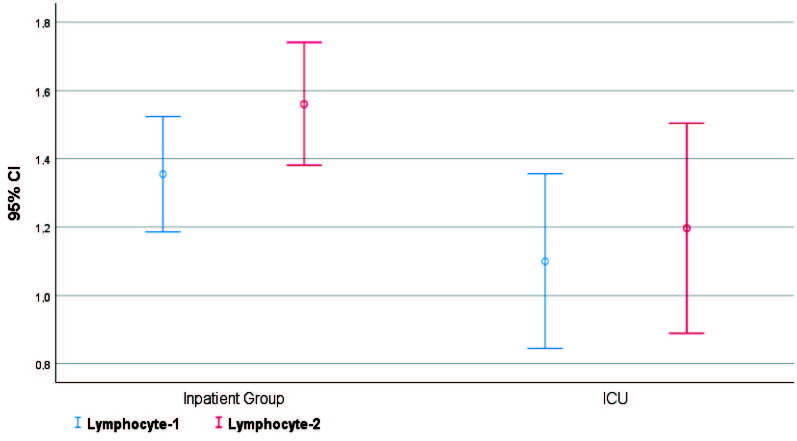
In-group and intergroup comparisons/lymphocyte-1 and 2.

**Figure 4:**
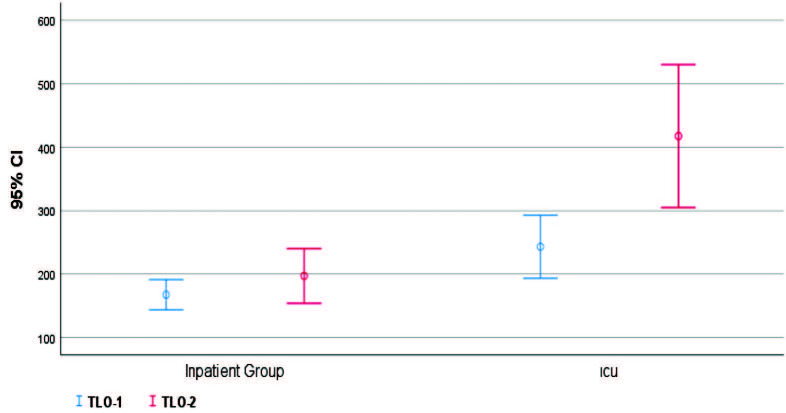
In-group and intergroup comparisons/TLO-1 and TLO-2.

**Figure 5:**
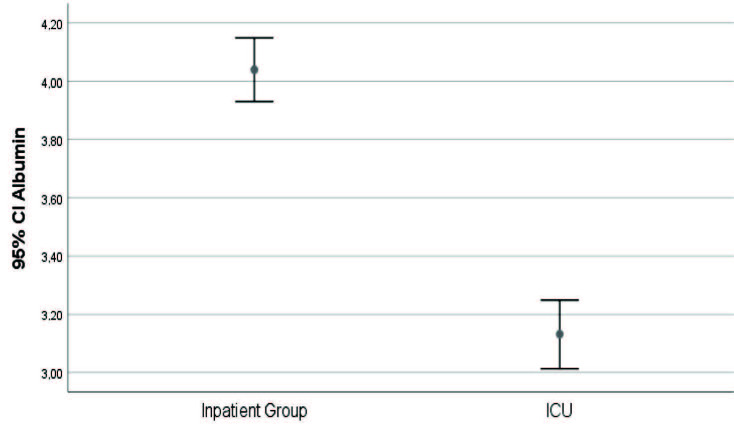
In-group and intergroup comparisons-serum albumin value.

Patients followed up in the ICU (severe COVID-19 patients) were found to be older, had higher rates of comorbidity, lower lymphocyte I and II values, higher TLO 1-2, and lower serum albumin levels (P < 0.05) (Figures 1–5). 

When the patients were evaluated in terms of thrombocyte, lymphocyte, and TLO values within groups; there was a statistically significant difference between thrombocyte 1 and 2 values in both ICU and inpatient unit patients (P < 0.05). It was found that the values at the second measurement time were higher in both groups. In lymphocyte values, there was no statistically significant difference between the first and second measurement values of patients in the ICU group (P > 0.05). At the same time, it was found that there was a statistically significant difference between the first and second measurement values of patients in the inpatient unit (P < 0.05), and the values at the second measurement time were high. In TLO values, there was no statistically significant difference between the first and second measurement values of patients in the inpatient unit group (P > 0.05). At the same time, it was found that there was a statistically significant difference between the first and second measurement values of patients in ICU (P < 0.05), and the values at the second measurement time were high. 

**Figure 6:**
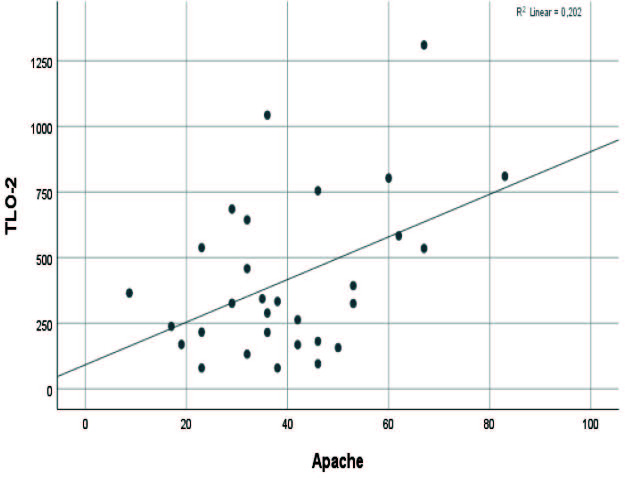
Relations with Apache–TLO-2.

When serum albumin level and thrombocyte/lymphocyte ratios were evaluated with acute physiology and chronic health evaluation II (APACHE II) of patients admitted to intensive care due to severe COVID-19 disease; it was found that there was a positive correlation between TLO-2 values and apache values at the level of r = 0.450 and this relationship was statistically significant (P < 0.05). (Figure 6).

**Figure 7:**
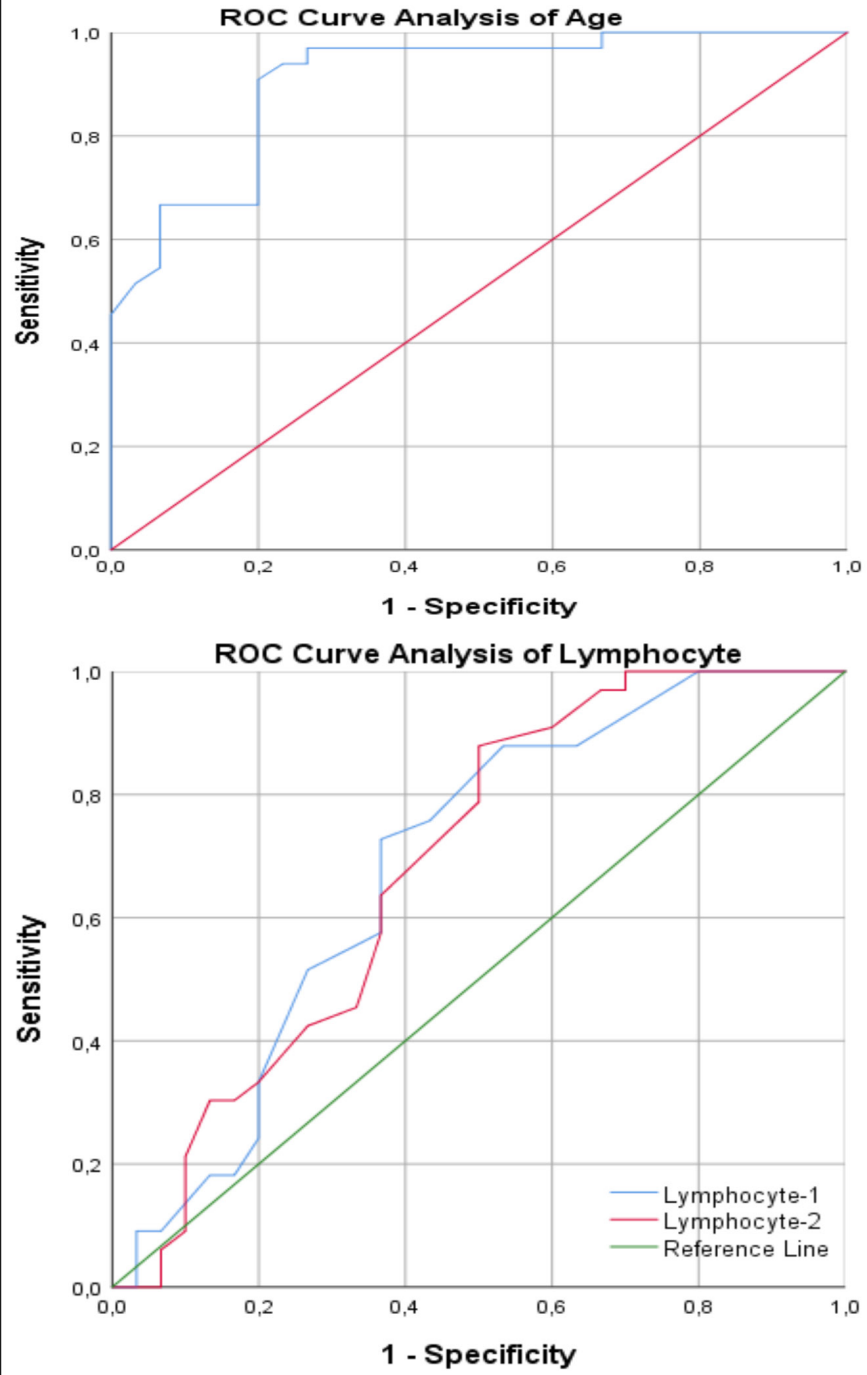

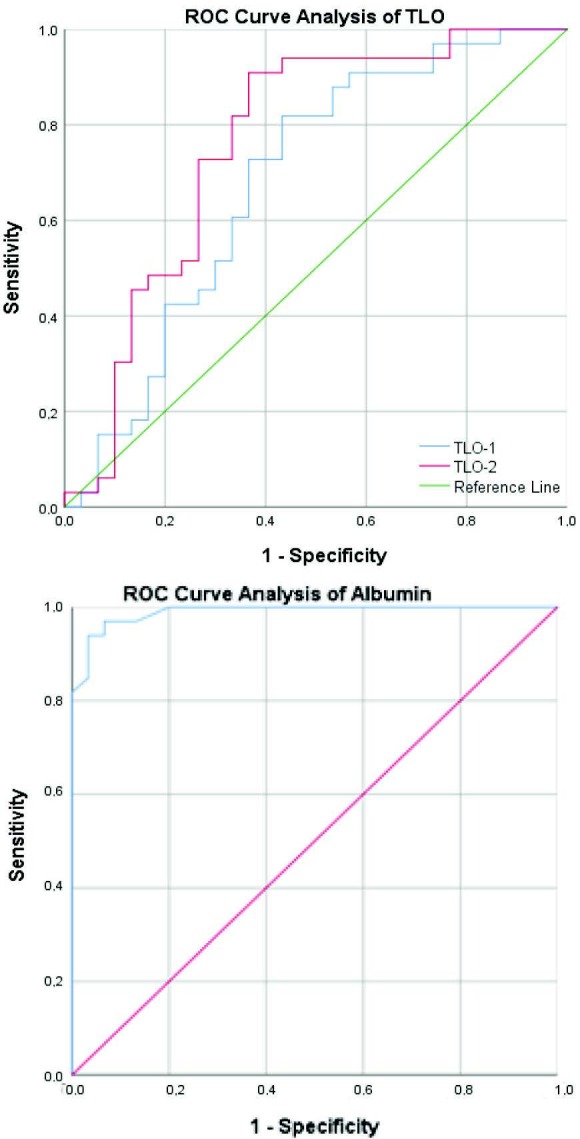
Recommended cut-off values for important parameters in inpatient group - ICU hospitalization.

It was found that the relationship between serum albumin during ICU and APACHE values at ICU admission was not statistically significant (P > 0.05) (Table 3). As a result of the evaluations made with the ROC analysis, when the service-ICU groups were compared in terms of the need for intensive care follow-up; age values > 54 were found to be the cut-off point (AUC = 0.906, P = < 0.0001, 95% CI: 0.806 to 0.965). It was found that 1 103 cells /µL was the cut-off point in lymphocyte-1 values (AUC = 0.684, P = 0.0084, 95% CI: 0.554–0.795). It was found that ≤ 1 103 cells/µL was the cut-off point in lymphocyte-2 values (AUC = 0.683, P = 0.009, 95% CI: 0.553–0.794). It was found that > 221 was the cut-off point in TLO-1 values (AUC = 0.681, P = 0.0101, 95% CI: 0.551–0.793). TLO-2 values > 260 were found to be the cut-off point (AUC = 0.762, P = 0.0001, 95% CI: 0.638–0.860). It was found that 3.6 g/dL was the cut-off point for albumin values (AUC = 0.989, P = < 0.0001, 95% CI: 0.924–1.000) (Table 4, Figure 7).

**Table 3:** Relations with Apache.

	Apache	
	r	P*
Albumin	–0.130	0.494
TLO-1	0.149	0.432
TLO-2	0.450	0.013

**Table 4:** Recommended cut-off values for important parameters in inpatient-ICU hospitalization.

	AUC	Cut-off	Sensitivity	Specificity	%95 GA	P*
Age	0.906	>54	80.0	90.9	0.806–0.965	<0.001
Lymphocyte-1	0.684	≤1	63.3	72.7	0.554–0.795	0.008
Lymphocyte-2	0.683	≤1	50.0	87.9	0.553–0.794	0.009
TLO-1	0.681	>221	56.7	81.8	0.551–0.793	0.010
TLO-2	0.762	>260	63.3	90.9	0.638–0.860	<0.001
Albumin	0.989	≤3.6	96.7	93.9	0.924–1.000	<0.001

## 4. Discussion

There is currently no specific treatment for COVID-19. Therefore, evaluation of the severity of disease and prognosis becomes increasingly essential [5]. Plebani et al. stated that laboratory data could play an essential role in the early detection, diagnosis, and management of many diseases [6]. Thrombocytes have important roles in coagulation and inflammatory pathways. Impairments in their number and functionality may closely be related to various diseases [7,8]. Thrombocytes are produced by megakaryocytes in the bone marrow. Such as inflammation: IL-6 can stimulate the formation of megakaryocytes by increasing the level of TPO [9,10]. In their analysis of 138 patients, Wang D et al. found that the total number of white blood cells in the peripheral blood of these patients was normal or decreased in the early stage of the disease, and the number of lymphocytes also decreased [11]. In our study, when both groups were compared in terms of lymphocyte values, it was found that lymphocyte values in patients following the intensive care unit were lower than those followed in the inpatient unit.

In a retrospective study of 30 patients, Rong Qu et al. showed that the lymphocyte level at the first admission to the hospital was closely related to the prognosis. In our study, it was found that the lymphocyte II values of the patients who were followed up in the inpatient unit were higher than the patients followed in the intensive care unit due to severe COVID-19 disease. (P < 0.05). In addition, in the study of Rong Qu et al., patients with high TLO values during the treatment of COVID-19 had a long hospital stay, and severe pneumonia was developed in these patients [12]. As a result of evaluation with ROC analysis, they determined the TLO cut-off point as 126.7 and the sensitivity as 100%, and the specificity as 86%. In our study, we found that TLO-2 cut-off point was > 260, sensitivity 90%, and specificity 63.3% (P < 0.001). In addition, in our study, there was no statistically significant difference (P > 0.05) in TLO values between the first and second measurements in the inpatient unit group. In contrast, there was a statistically significant difference between the first and second measurement values in the ICU group (P < 0.05). The second measurement values were found to be higher. 

It was found that there was a positive correlation between TLO-2 values and acute physiology and chronic health evaluation II (APACHE II) values at the level of r = 0.450, and this relationship was statistically significant (P < 0.05). When this situation is evaluated in terms of mortality and morbidity for patients who need intensive care follow-up, TLO-2 values give clinicians a meaningful idea about the disease’s course and prognosis during their follow-up. 

Huang et al. evaluated the data of 36 patients who died due to COVID-19 in 2020 and reported that 80.65% of patients had hypoalbuminemia and 70.59% lymphopenia [13].

Albumin is a protein synthesized by the liver that plays an important role in maintaining nutrition and plasma osmolarity [14]. Juyi Yi et al. stated that low levels of albumin were an indicator of poor nutritional status of the patient and this condition also decreased the body’s immunity, and the host’s immune response to RNA virus infection was often weakened by nutritional deficiencies that could be ignored during clinical diagnosis and treatment [15]. In this study, Juyi Yi et al. determined the cut-off point of serum albumin level in confirmed COVID-19 critically ill patients as 3.51 g/dL (sensitivity: 76.47%; specificity: 73.81%) [15]. Similarly, in our study, we determined the cut-off point of serum albumin value in critically ill patients as ≤ 3.6 g/dL. (sensitivity: 96.7; specificity: 93.9).

Liu Y. et al. noted that the most common laboratory abnormalities were hypoalbuminemia, lymphopenia, decreased lymphocyte and neutrophil percentage, high C-reactive protein (CRP), high lactate dehydrogenase (LDH), and decreased CD8 count. They also found that serum albumin value, lymphocyte cell count and percentage, neutrophil percentage, lactate dehydrogenase, and CRP levels were highly correlated with acute lung injury [16]. This situation could be critical in the patient group with severe COVID-19 disease.

Liu W. et al. examined 78 patients with COVID-19 PCR +. They found that serum albumin level was significantly lower in the clinically progressed patient group compared to the clinically stable patient group (36.62 ± 6.60, 41.27 ± 4.55 g/dL, U = 2.843, P = 0.006) [17]. In our study, we also found low serum albumin levels in patients with severe COVID-19 (3.1 ± 0.2, 4.0 ± 0.3 g/dL, P = 0.000). 

## 5. Conclusion

The TLO-2 ratio above 260, the lymphocyte level below 1 103 cells/µL, and the albumin value below 3.6 g/dL would be a predictor of further intensive care unit need and might suggest that the patient’s clinic would be severe. Patients with these laboratory values should be followed closely and acted upon quickly during the treatment process.
